# E-cigarette liquids alter the biogenesis and properties of *Aggregatibacter actinomycetemcomitans* outer membrane vesicles

**DOI:** 10.3389/fcimb.2026.1731156

**Published:** 2026-02-10

**Authors:** Mahsa Khodabakhsh Majd, Lillian Y. Wu, Giancarlo A. Cuadra, Angela C. Brown

**Affiliations:** 1 Department of Chemical and Biomolecular Engineering, Lehigh University, Bethlehem, PA, United States; 2 Department of Biology, Muhlenberg College, Allentown, PA, United States

**Keywords:** *A. actinomycetemcomitans*, electronic cigarettes, outer membrane vesicles, flavored E-cigarette liquid, E-liquids

## Abstract

*Aggregatibacter actinomycetemcomitans* is an oral pathogen associated with aggressive forms of periodontitis as well as systemic diseases such as endocarditis. Through the secretion of outer membrane vesicles (OMVs), this bacterium establishes complex host–pathogen interactions, transmitting virulence determinants such as leukotoxin A (LtxA) that influence host cell behavior. Smoking of both traditional cigarettes as well as electronic cigarettes (ECIG) has been correlated with increased rates of periodontitis, yet the mechanisms regulating this have not been fully elucidated. We hypothesized that ECIG liquids (E-liquids), which contain numerous organic molecules, including nicotine and flavoring agents, might affect *A. actinomycetemcomitans* virulence by altering OMV production. To test this, we examined the impact of cinnamon and menthol flavored E-liquid exposure on *A. actinomycetemcomitans* growth as well as the production and composition of the resulting OMVs. Both cinnamon and menthol E-liquids inhibited *A. actinomycetemcomitans* growth at relatively low concentrations, with cinnamon E-liquid exhibiting greater toxicity than menthol. Bacteria treated with subinhibitory concentrations of the E-liquids produced fewer OMVs than untreated cells. While the average OMV size remained similar between treated and untreated cells, cinnamon E-liquid exposure shifted OMV production toward larger vesicles. Cinnamon E-liquid exposure also increased the protein and surface-associated DNA concentrations of the OMVs. SDS-PAGE showed significant differences in the protein composition between OMVs produced by untreated or treated bacteria, but no changes in membrane fluidity were detected among the OMV groups. Together, these results demonstrate that E-liquids modulate OMV biogenesis and composition in *A. actinomycetemcomitans*, potentially influencing its virulence and pathogenicity.

## 1 Introduction

Originally, electronic cigarettes (ECIGs) were intended as a “harm-reduction” means for cessation of traditional smoking. However, ECIGs have now become a multimillion-dollar industry similar to that of “Big Tobacco” (Cahn and Siegel, 2011; Palazzolo, 2013). Although vaping is advertised as a safer alternative to smoking, the effects of inhaling ECIG-generated aerosols have not yet been fully explored (Foulds and Veldheer, 2011; Foulds et al., 2011). The liquid within the cartridge of an electronic cigarette is commonly known as E-liquid and consists of the base humectants, nicotine (ranging from 0 to 24 mg/mL), and flavoring agents which comprises up to 25% of the E-liquid (Krüsemann et al., 2018; Fischman et al., 2020). E-liquid aerosol particles range in size from <0.3 µm to >10 µm (Palazzolo et al., 2021). The biological effects of the particles depend largely on their deposition in the mouth and in the respiratory tract, being highly dependent on the manner of integration into biological fluids and tissues (Sosnowski and Odziomek, 2018). To date, most of the scientific literature on vaping focuses on the effects on the airway (Allbright et al., 2024; Worden et al., 2024; Yang et al., 2025), but little attention has been paid to the oral microenvironment.

The oral cavity contains 774 species of microbes including commensal and pathogenic bacteria (HOMD:: Human Oral Microbiome Database). Under healthy conditions, these microbes form communities commonly known as dental plaque, which are biofilms formed on tooth surfaces. Dysbiosis is the lack of balance between commensals and pathogens, leading to anomalies such as periodontal disease. This disease is the result of chronic inflammation in the tissues surrounding and supporting the teeth leading to tooth loss as well as systemic complications (Beck et al., 1996; Garcia et al., 2001; Hajishengallis and Lamont, 2012; Borgnakke et al., 2013; Singhrao et al., 2015; Daalderop et al., 2018). *Aggregatibacter actinomycetemcomitans* is a Gram-negative bacterium and one of the key pathogens associated with the onset and development of periodontal disease.

The primary virulence factor of *A. actinomycetemcomitans* is a leukotoxin (LtxA) that specifically kills human immune cells (Mangan et al., 1991; Johansson, 2011). Strains of *A. actinomycetemcomitans* that produce more LtxA, such as the JP2 clone, are more closely associated with disease progression, indicating an important role for this toxin in disease (Zambon et al., 1983; Haraszathy et al., 2000; Haubek et al., 2008). LtxA has been shown to be released into the extracellular environment in a “free” form (as a soluble protein), as well as in association with outer membrane vesicles (OMVs) (Kato et al., 2002; Nice et al., 2018), nanoscale spherical vesicles derived from the Gram-negative bacterial outer membrane (Kuehn and Kesty, 2005), which enable the long-distance delivery of these proteins to host cells (Bomberger et al., 2009). Recently, specific links between Alzheimer’s Disease (AD) and oral infectious agents, including *A. actinomycetemcomitans* have been reported (Díaz-Zúñiga et al., 2019; Dominy et al., 2019; Ha et al., 2020). Importantly, we and others have hypothesized that OMVs released during the periodontal infection promote the transport of LtxA and other cargo to the brain, initiating the pathogenic cascade associated with AD (Han et al., 2019; Gong et al., 2022; Butler et al., 2024). In other words, periodontal disease affects not just oral health, but neurological and systemic health as well.

The production of OMVs has been reported to be an envelope stress response to counter numerous environmental stresses, including temperature, antibiotics, and antimicrobial peptides (McBroom and Kuehn, 2007; MacDonald and Kuehn, 2013). For example, several antibiotics, including ciprofloxacin, meropenem, fosfomycin, and polymyxin B were found to increase OMV production in two strains of *Escherichia coli* when present in sub-inhibitory concentrations (Bauwens et al., 2017). Multiple studies have found that OMV production is increased in response to the presence of antimicrobial peptides, such as LL-37 and polymyxin B (Manning and Kuehn, 2011; Urashima et al., 2017). Importantly, the protein composition, in particular the virulence factor concentration, of the produced OMVs has been reported to change in response to stress conditions (Klimentova et al., 2019; McMillan Hannah and Kuehn Meta, 2022), potentially altering the immunomodulatory behavior of the vesicles. We therefore hypothesized that exposure to subinhibitory concentrations of E-liquids could affect bacterial pathogenesis through alteration of OMV physical and biochemical properties. In this project, we investigated the effects of cinnamon- and menthol-flavored E-liquids on the number and size of OMVs produced by *A. actinomycetemcomitans*, as well as their protein concentration and composition, surface-associated DNA levels, and membrane fluidity.

## 2 Materials and methods

### 2.1 Preparation of E-liquids

E-liquids were prepared as previously described (Fischman et al., 2020; Xu et al., 2022; Christian et al., 2024). Briefly, propylene glycol and vegetable glycerol were mixed at a 1:1 ratio (solvent) and spiked with 20 mg/mL nicotine and 5% v/v cinnamon or menthol flavors. E-liquids were stored at 4°C and diluted in *A. actinomycetemcomitans* growth medium (AAGM, 3 g trypticase soy broth and 0.6 g yeast extract in 100 mL DI water) for experiments.

### 2.2 Growth of A. actinomycetemcomitans with and without E-liquids

*A. actinomycetemcomitans* (JP2 genotype, a gift from Dr. Edward T. Lally) was initially cultured as previously described (Kachlany et al., 2002). Briefly, bacteria were grown in 100 mL AAGM (3 g trypticase soy broth and 0.6 g yeast extract), along with 5 µg vancomycin, 75 µg bacitracin, 0.8 g dextrose, and 4 mL 10% sodium bicarbonate (Tsuzukibashi et al., 2008) in a candle jar, at 37°C with no shaking for 18 hr.

For the growth curve experiments, the starter culture was diluted to an optical density (OD_600_) of 0.06 in a 96-well plate, along with menthol or cinnamon E-liquids in a range of concentrations. The plate was incubated at 37°C, and the OD_600_ was recorded hourly using a Tecan plate reader. The change in OD_600_ (ΔOD_600_), relative to the initial reading was calculated hourly.

### 2.3 Collection of OMVs

Starter cultures were diluted 6:100 in a larger volume of AAGM (with vancomycin, bacitracin, dextrose, and sodium bicarbonate) along with menthol or cinnamon E-liquid and then cultured for another 24 hrs at 37°C with no shaking. OMVs were collected from the bacterial supernatants following a published protocol (Kato et al., 2002). Briefly, bacterial cultures were centrifuged twice at 10, 000 × g for 10 min to pellet the cells. Supernatants were filtered using a 0.45-µm filter and then concentrated using 50-kDa molecular weight cutoff (MWCO) centrifugal filters. Filtered supernatants were ultracentrifuged twice at 105, 000 x g and 4°C for 30 min. Pellets, containing the OMVs, were resuspended in 2 ml of Tris buffer (150 mM NaCl, 10 mM Tris, pH 7.0). Finally, the OMV suspensions were centrifuged for 5 min at 5, 000 x g to remove any remaining debris, and the supernatant was then filtered through a 0.45-μm syringe membrane filter (ThermoFisher Scientific, polyethersulfone (PES) membrane). The purified OMVs were stored at −20°C until use (Kato et al., 2002).

The lipophilic FM™ 4–64 dye (ThermoFisher Scientific) was used to determine the relative lipid concentrations of the purified OMVs (MacDonald and Kuehn, 2013). Liposomes composed of 1-palmitoyl-2-oleoyl-glycero-3-phosphocholine (POPC) were prepared and diluted serially. The FM™ 4–64 dye (5 μg/L) was added to the serially diluted liposomes as well as to serially diluted OMV solutions. The fluorescence intensity (excitation: 515 nm, emission 640 nm, collected using a Tecan plate reader) of the OMV solution was compared to that of a calibration curve created using the POPC liposomes to determine the lipid concentration of the OMVs, as previously described (MacDonald and Kuehn, 2013).

To determine the amount of protein in each OMV sample, the absorbance at 280 nm (A_280_) was measured using a NanoDrop spectrophotometer (ThermoFisher Scientific) (Simonian and Smith, 2006).

### 2.4 Liposome synthesis

The thin film method was used to synthesize liposomes (Zhang, 2017). POPC (25 mg/mL in chloroform) was added to a glass vial in the required amount. The chloroform was then evaporated using a stream of air to leave a thin lipid film. The lipid film was placed under vacuum overnight to remove any residual chloroform. Tris buffer was added to the films, which were then vortexed and extruded 13 times through 100 nm polycarbonate filters to create unilamellar liposomes.

### 2.5 Dynamic light scattering

The size of the OMVs was determined by dynamic light scattering (DLS). Samples were diluted 1:20 in Tris buffer and passed through a 0.45-µm pore-size PES membrane to minimize contamination. Measurements were performed on an ALV/CGS-3 compact goniometer at 632.8 nm wavelength, with a 90° scattering angle and a run time of 180 s. Each sample was analyzed in triplicate. Data acquisition and processing were carried out using the ALV proprietary software, which generated the number weighted size distribution using a regularized fit and assuming a membrane thickness of 5 nm (r* = 5 nm) (Nagle and Tristram-Nagle, 2000; Pencer and Hallett, 2003).

### 2.6 TOTO-1 fluorescence staining

The DNA-intercalating dye TOTO-1 (ThermoFisher Scientific) was used to assess the relative surface-associated DNA levels of the purified OMVs (Hirons et al., 1994). OMVs were diluted to a final lipid concentration of 0.2 mM and mixed with an equal volume with TOTO-1 dye. The samples were incubated under dark conditions prior to fluorescence measurements for 15 minutes. The fluorescence was measured using a PTI fluorescence spectrometer [Photon Technology Inc. (PTI)] with excitation at 514 nm and emission collected at 533 nm.

### 2.7 Laurdan fluorescence staining

Laurdan fluorescence was used to assess the membrane fluidity of purified OMVs. OMVs were prepared at a lipid concentration of 1 mM in Tris buffer and incubated with Laurdan (100 µM, 1:5) at 37 °C for 2 h. Following incubation, the samples were centrifuged using Millipore 30 kDa molecular weight cutoff membrane filters to remove excess unbound dye, washed, and resuspended in fresh Tris buffer. For fluorescence measurements, 100 µL of each sample was transferred to a black 96-well plate, and fluorescence intensity was measured using a Tecan plate reader (excitation: 368 nm; emission: 400–500 nm). The resulting spectra were normalized to the maximum emission intensity of each sample. The Laurdan emission spectrum exhibits two characteristic peaks corresponding to distinct membrane states; the emission at 440 nm (I_440_) represents the gel (ordered) state, while the emission at 490 nm (I_490_) represents the liquid-crystalline (disordered) state. Generalized polarization (GP) values were calculated using Equation 1, where higher GP values indicate lower membrane fluidity (Parasassi et al., 1991).

(1) $$\frac{I_{440}-I_{490}}{I_{440}+I_{490}}$$

### 2.8 SDS-PAGE and protein staining

Control and treated *A. actinomycetemcomitans* OMV samples were collected as above, lyophilized and then resuspended in 20 µL water to measure protein concentration using the NanoDrop. Based on protein concentrations, 100 µg of each sample were added to a mixture of 100 µL final volume, containing SDS sample buffer and 2.5 µM dithiothreiotol to reduce disulfide bonds. Samples were denatured at 100°C for ten minutes. After denaturing and reducing, 40 µg of each protein sample were loaded and separated in a 4% - 20% gradient gel (GenScript Biotech, Piscataway, NJ, USA). The electrophoresis settings were 140 V, 80 mA and 150 Watts for 1 hour and then reduced to 100 V, 50 mA and 100 Watts for 45 minutes. Gels were then washed and stained in Coomassie overnight. For silver nitrate enhancement, the gel was stained as previously established (Merril et al., 1982). Bands were visualized using the ChemiDoc MP Imaging System (Bio-Rad Laboratories Inc. Philadelphia, PA, USA). Band densities were quantified using ImageJ (v. 1.54g), and the consistent 35 kDa bands were used as a loading control to normalize protein percentages for each treatment or control.

### 2.9 Statistical analysis

Statistical analyses were performed using MATLAB (MathWorks, Natick, MA, USA). Comparisons between two independent groups were conducted using a two-tailed Welch’s two-sample t-test to account for potential unequal variances and unequal sample sizes. Summary statistics (mean, standard deviation, and sample size) were used to compute the t-statistic, degrees of freedom based on the Welch–Satterthwaite approximation, and corresponding p-values. Results were considered statistically significant at p < 0.05.

## 3 Results

### 3.1 E-liquids inhibit bacterial growth and reduce OMV production

**Figure 1:**
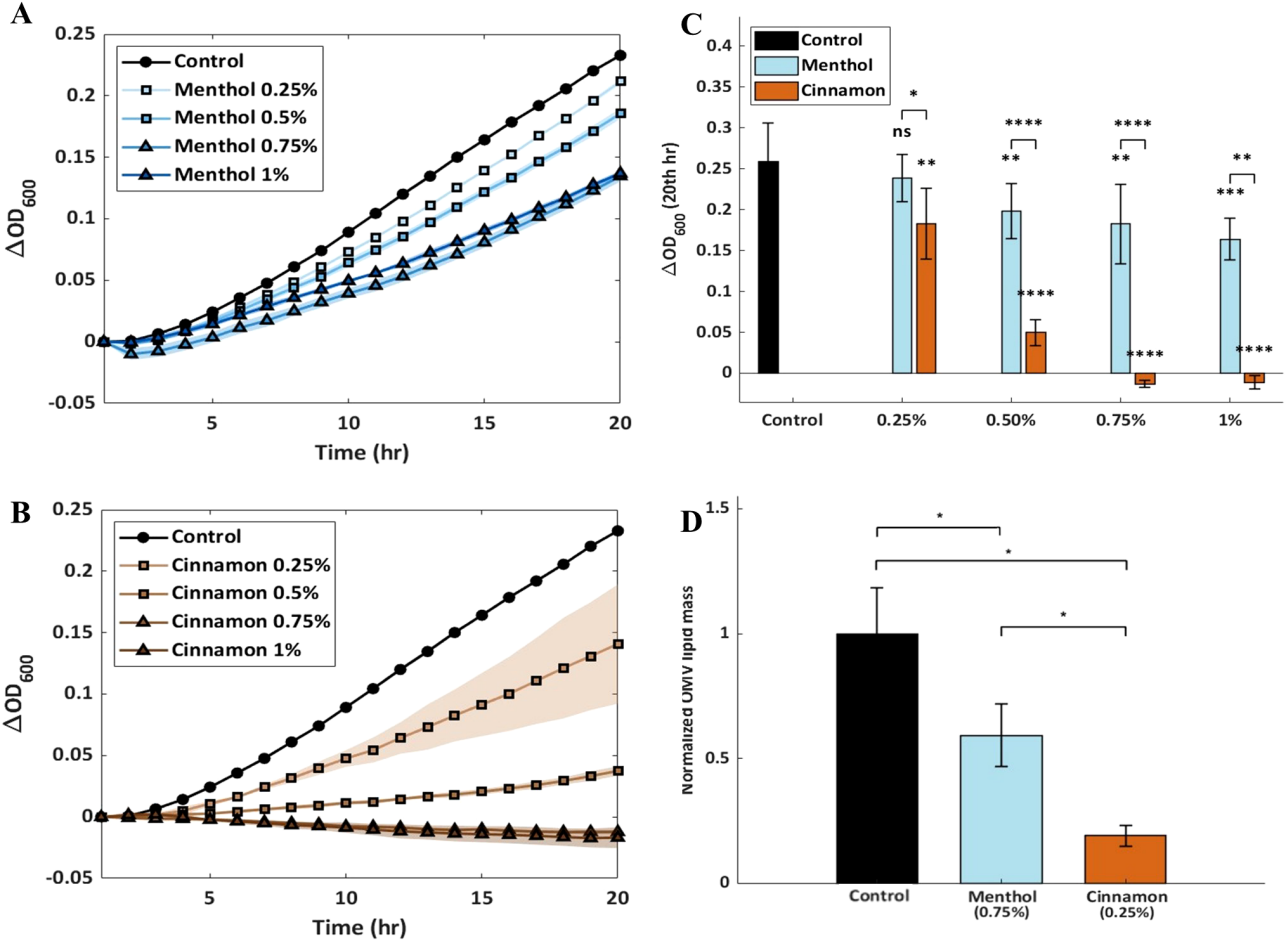
Effect of menthol and cinnamon E-liquids on *A. actinomycetemcomitans* growth and OMV production. Growth of *A. actinomycetemcomitans* over 20 hours in AAGM supplemented with menthol-flavored **(A)** or cinnamon-flavored **(B)** E-liquid at four concentrations. Each data point represents the mean of three technical replicates, with shading indicating the standard deviation. **(C)** Change in OD_600_ after 20 hours across all treatment groups. Each data point represents the mean of two biological replicates, each measured three times (n = 6), with error bars showing the standard deviation. *p<0.05; **p<0.01; ***p<0.001; ****p<0.0001. **(D)** Lipid mass of OMVs purified from *A. actinomycetemcomitans* cultures grown in AAGM (control) or AAGM supplemented with 0.25% cinnamon or 0.75% menthol E-liquid. Each value was normalized to the lipid mass of the untreated control sample. Each data point represents the mean of three biological replicates, each measured three times, with error bars showing the standard deviation. *p < 0.05.

The effect of menthol- or cinnamon-flavored E-liquids on *A. actinomycetemcomitans* growth was determined by measuring *A. actinomycetemcomitans* growth in AAGM alone (untreated) or AAGM supplemented with the E-liquids at four concentrations (0.25%, 0.5%, 0.75%, and 1%). As shown in **Figure 1**, both E-liquid treatments inhibited bacterial growth, with menthol E-liquid treatment (**Figure 1A**) inhibiting growth at concentrations above 0.5% and cinnamon E-liquid treatment (**Figure 1B**) inhibiting growth at all tested concentrations. The cinnamon E-liquid exhibited a stronger inhibitory effect than menthol E-liquid; concentrations greater than 0.5% led to complete growth suppression. Based on these findings, media containing 0.75% menthol or 0.25% cinnamon E-liquids were selected for further experiments on OMV production, as these conditions produced similar levels of growth inhibition while remaining clearly distinct from the untreated control (**Figure 1C** ).

Next, we investigated the effect of menthol and cinnamon flavored E-liquids on OMV production. **Figure 1D** shows the relative lipid mass of OMVs collected from *A. actinomycetemcomitans* grown in AAGM (control) or AAGM supplemented with 0.75% menthol or 0.25% cinnamon E-liquids. Both E-liquids reduced OMV production relative to the control, with cinnamon treatment causing the strongest effect, an approximately five-fold reduction. All OMV-related measurements presented here are reported as relative comparisons across experimental conditions, enabling evaluation of treatment-dependent trends despite the absence of quantitative particle counting.

### 3.2 E-liquid exposure has minimal effect on OMV size

**Figure 2:**
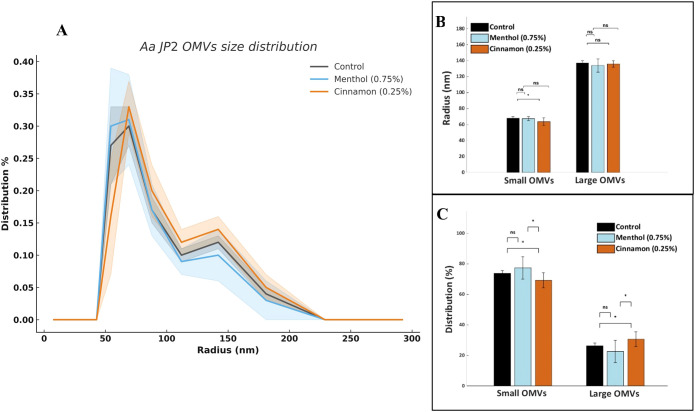
OMV size distributions measured by DLS. **(A)** Distributions of OMV radii corresponding to the major OMV populations produced by *A. actinomycetemcomitans.* Each data point represents the mean of three biological replicates, each measured in triplicate. Shading indicates the standard deviation. **(B)** Comparison of the radii of small (<112 nm) and large (>112 nm) OMVs produced by untreated (control) or menthol or cinnamon E-liquid–treated bacteria. Data are presented as the mean ± standard deviation of three biological replicates, each measured in triplicate. NS, not significant; *p<0.05. **(C)** Distribution of small and large OMVs expressed as percentages of the total OMV population. Data are presented as the mean ± standard deviation of three biological replicates, each measured in triplicate. NS, not significant; *p<0.05.

Prior work in our lab has found that *A. actinomycetemcomitans* JP2 releases two populations of OMVs, one highly abundant population of small OMVs (radius < 112 nm), and a second population of larger OMVs (radius > 112 nm). Importantly the leukotoxin (LtxA) concentration of the OMV populations differs, with LtxA being predominately located on the larger OMVs (Collins et al., 2021; Nice et al., 2024; Singh et al., 2024). Here, we evaluated the effect of E-liquids on OMV size distributions using DLS. We observed similar overall OMV size distributions across all three treatments (**Figure 2A**). Comparison of the average size of “small” and “large” OMVs indicated a modest but significant decrease in size of small OMVs from the cinnamon E-liquid-treated cells relative to the control (**Figure 2B**). In terms of relative proportions, cinnamon E-liquid-treated bacteria produced fewer small OMVs and more large OMVs compared with both the menthol-treated and control groups (**Figure 2C**).

### 3.3 E-liquid exposure causes significant changes in OMV protein and DNA concentration

**Figure 3:**
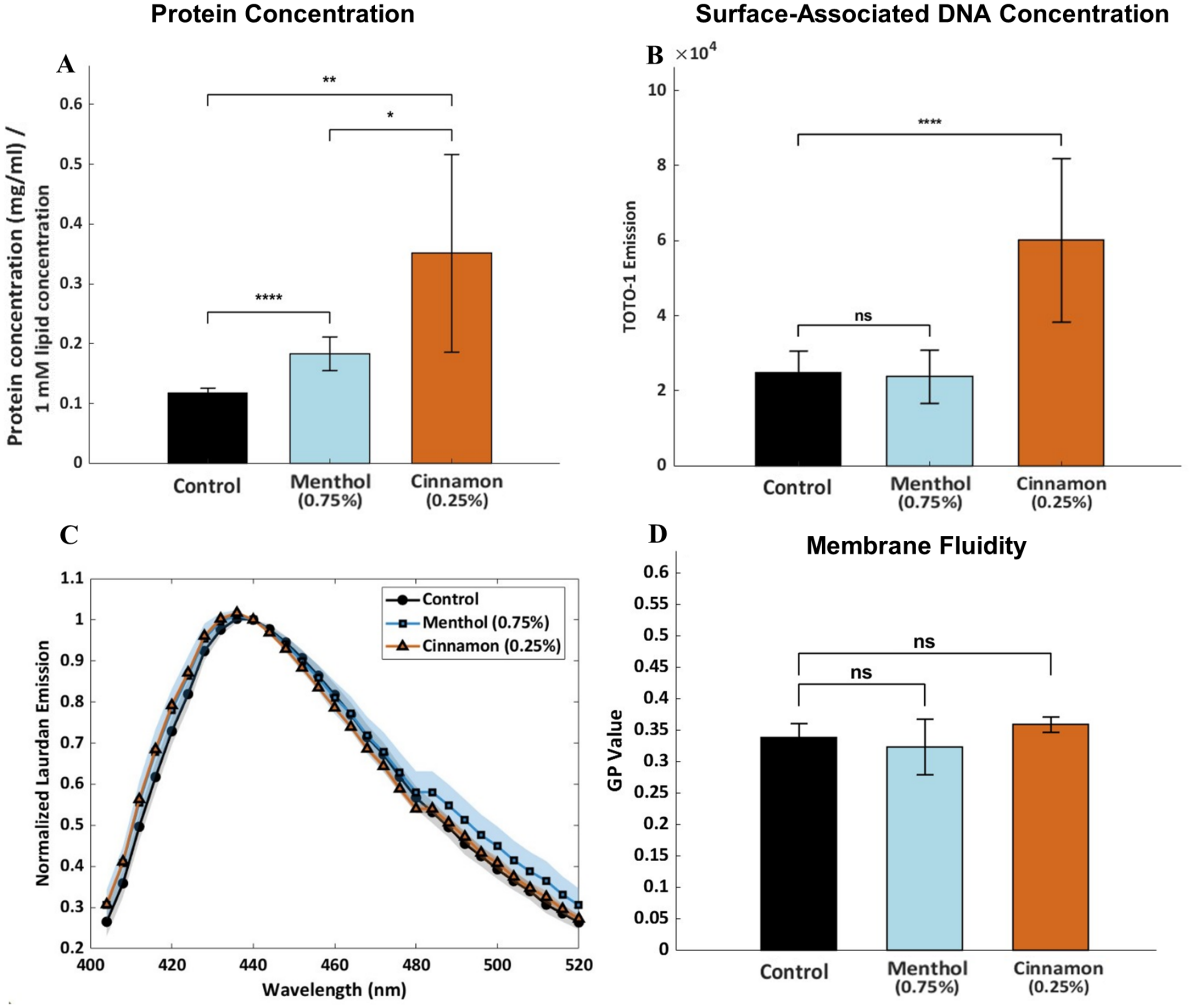
Protein and DNA Composition and Fluidity of OMVs **(A)** Protein concentration of OMVs collected from *A. actinomycetemcomitans* cultures. Each culture was grown in AAGM (control) or AAGM supplemented with menthol or cinnamon E-liquid. The OMV protein concentration of each sample was normalized to 1 mL lipid concentration to account for differences in OMV production amounts between treatments. Each data point represents the mean of three biological replicates, each measured three times, with error bars showing the standard deviation. *p<0.05, **p<0.01, ***p<0.001. **(B)** TOTO-1 fluorescence emission of OMVs from the three treatment groups, reflecting the relative amount of surface-associated DNA. Each data point represents six measurements obtained from samples of similar concentration, with error bars showing the standard deviation. Statistical significance is denoted as follows: NS, not significant; p < 0.0001 (****). **(C)** Normalized Laurdan emission spectra illustrating differences in OMV membrane fluidity. Shaded regions represent the standard deviation across 6 replicates. **(D)** GP values calculated from six independent measurements, demonstrating no statistically significant differences in membrane fluidity among the groups.

The protein concentration of the OMVs was measured using a NanoDrop spectrophotometer and normalized to the molar lipid concentration of each preparation. The resulting protein content per 1 mM lipid is shown in **Figure 3A**. OMVs collected from both menthol and cinnamon E-liquid-treated cultures had significantly higher protein concentrations than untreated control samples. The OMVs collected from the cinnamon-supplemented culture exhibited the highest protein-to-lipid ratio, exceeding both control and menthol-treated groups.

TOTO-1 fluorescence staining was used to assess relative surface-associated DNA levels among the OMV samples. OMVs derived from cinnamon E-liquid–treated cultures (**Figure 3B**) exhibited relatively higher TOTO-1 fluorescence compared to the other groups, indicating increased surface-associated DNA. In contrast, OMVs derived from menthol E-liquid-treated cultures contained a similar amount of surface-associated DNA as the untreated control.

To determine if large-scale changes in lipid composition occur upon E-liquid exposure, we used Laurdan fluorescence to determine changes in membrane fluidity. **Figure 3C** shows the resulting normalized fluorescence spectra revealing no significant differences in membrane fluidity among the groups. The GP values, which indicate membrane fluidity, with lower values indicating more gel-like membranes, are shown in **Figure 3D** and also indicate no significant differences.

### 3.4 E-liquid exposure changes protein profile in OMVs

**Figure 4:**
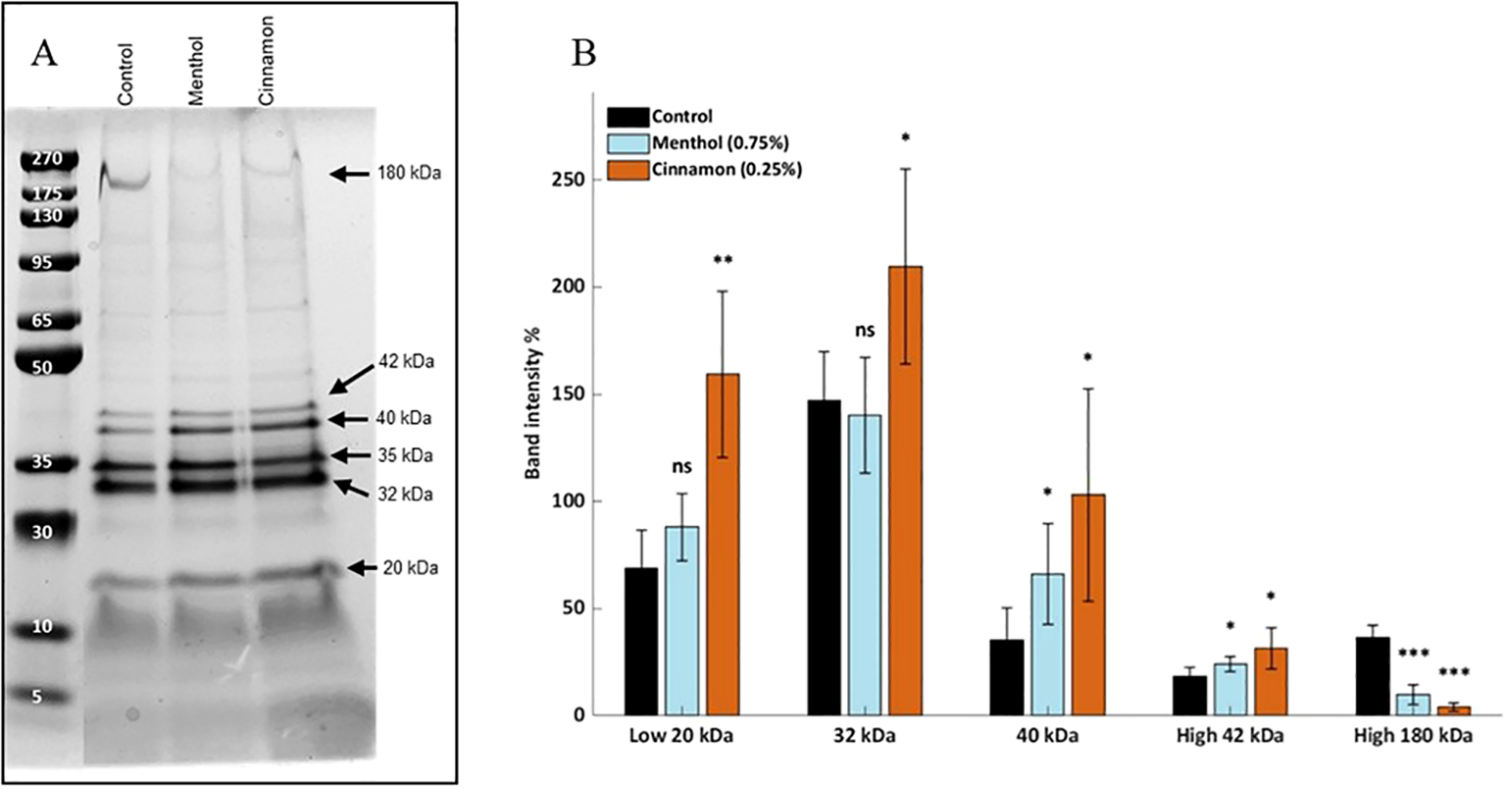
SDS-PAGE of *A. actinomycetemcomitans* OMV proteome. **(A)** Each culture was grown in AAGM (untreated control) or AAGM supplemented with menthol or cinnamon E-liquid. OMVs were collected, lyophilized and separated by SDS-PAGE. The gel was stained with Coomassie blue and enhanced with silver nitrate. Representative image from two separate trials. **(B)** Quantification of the differentially expressed bands, each normalized to the intensity of the 35 kDa band. NS, not significant; *p<0.05, **p<0.01, ***p<0.001, relative to the untreated control (n = 4, two biological x two technical replicates).

Separation and Coomassie/silver staining of OMV proteins via SDS-PAGE revealed differences in the expression of several proteins within the *A. actinomycetemcomitans* OMVs between control and E-liquid treatments (**Figure 4A**). We observed that the 35 kDa molecular weight band was consistent across all OMV preparations. This band is most likely OmpA which is commonly used to normalize OMV concentration (Ojima et al., 2018) because of its consistent expression and localization in OMVs. We therefore used this band as a “loading control” and used it to determine the relative expression of other bands, including the 20 kDa, 32 kDa, 40 kDa, 42 kDa, and 180 kDa bands (**Figure 4B**). This analysis demonstrated that the 20, 32, 40, and 42 kDa bands increased in intensity in OMVs produced by cinnamon-treated cultures, while that of the 120 kDa band decreased. In OMVs produced by menthol-treated cultures, the 40 and 42 kDa bands increased and the 180 kDa band decreased. Altogether, the data indicate that E-liquids induce differential OMV proteome patterns, visible without an extremely sensitive assay (i.e., Western blot).

## 4 Discussion

ECIGs are often advertised as safer alternatives to traditional tobacco products, but our results indicate that the components found in the E-liquids may have significant effects on oral health. Exposure to flavored E-liquids can significantly influence *A. actinomycetemcomitans* bacterial growth and OMV production in a flavor-dependent manner. Specifically, cinnamon flavor exhibited stronger antimicrobial activity than menthol, with significant growth inhibition observed at concentrations as low as 0.25%. This observation is consistent with prior reports on the antimicrobial properties of cinnamaldehyde against oral pathogens (Wang et al., 2018; He et al., 2019; Panjaitan et al., 2022). Although less has been reported about the antibacterial activity of menthol, our study showed milder but significant inhibitory effects against *A. actinomycetemcomitans*. Flavorless E-liquid with or without nicotine has been shown to have little to no effects on oral bacterial growth as colony-forming units (Cuadra et al., 2019), planktonically (Nelson et al., 2019; Fischman et al., 2020) or in biofilms (Xu et al., 2022; Christian et al., 2024) and therefore, was not used in this study. Similarly, prior work demonstrated that cinnamon and menthol E-liquids inhibit the growth and biofilm formation of oral commensal bacteria, displaying a toxic effect (Fischman et al., 2020; Xu et al., 2022; Christian et al., 2024), which agrees with our results.

We observed marked reductions in OMV production following E-liquid treatment. Cinnamon exposure reduced OMV output by about 80% relative to untreated controls, while menthol treatment caused an approximately 40% reduction. Since OMVs play a central role in bacterial communication, biofilm formation, and virulence factor delivery (Kulp and Kuehn, 2010; Ma and Cao, 2021), these reductions suggest that E-liquid exposure could have significant implications on *A. actinomycetemcomitans* pathogenesis. Multiple reports have shown that stress can alter OMV production and properties (MacDonald and Kuehn, 2013; Klimentova et al., 2019; Mozaheb and Mingeot-Leclercq, 2020; Hosseini Zadeh et al., 2022), supporting the idea that chemical exposures, including flavorants, may broadly reshape vesiculation pathways.

Unpublished data indicate that 100 puffs of aerosol from an ECIG, delivered to 50 mL of growth medium, results in roughly 0.2% of aerosol materials dissolved in the media. This is comparable to the 0.25% cinnamon E-liquid dissolved in the *A. actinomycetemcomitans* growth media in this study. Therefore, the amount of aerosol material in 100 puffs is 100 mg (w/v). The oral cavity contains roughly 1 mL of saliva on its surface (Iorgulescu, 2009). Consequently, after 100 puffs, without salivating or swallowing saliva during that time, the oral cavity would have 100 mg/mL of aerosol in the saliva, which is 10% w/v. More realistically, with salivation and swallowing, the aerosol concentration in the saliva would be much lower. Thus, the majority of studies on E-cigarettes and oral microbes tend to focus on percentages between 0.1 to 2% (Cuadra et al., 2025).

To our knowledge, this is the first study that evaluates oral bacteria OMV characteristics after E-liquid treatments. Interestingly, while menthol treatment did not significantly alter OMV size distribution, cinnamon exposure slightly shifted the vesicle populations toward larger OMVs (**Figure 2A**). After measuring OMV total proteins as a function of total lipids, both E-liquids significantly increased the protein amount (**Figure 3A**) suggesting that these treatments alter the OMV proteome. Our SDS-PAGE results demonstrated significant differences in protein composition, especially a high molecular weight protein and several smaller proteins (**Figure 4**). Only the high molecular weight protein of ~180 kDa is downregulated, while the lower molecular-weight proteins are mostly upregulated after E-liquid treatments, thus explaining the results in **Figure 3C**. The scope of the present work was intentionally focused on evaluating relative changes in protein abundance independent of protein identity, enabling a global assessment of proteomic shifts in the OMVs due to E-liquid exposure. Protein-specific identification and validation are critical for mechanistic interpretation and will be the subject of future work.

In addition, we investigated changes in surface-associated DNA and found that cinnamon E-liquid exposure significantly increased the amount of DNA on the OMVs. As surface-associated DNA has been implicated in bacterial interactions with the host and microbial persistence (Bitto et al., 2017), this increase may suggest altered biological properties of OMVs produced under cinnamon exposure. We used Laurdan fluorescence, a measure of membrane fluidity, to investigate potential variations in lipid composition. No significant differences were observed among OMVs from control, menthol, and cinnamon E-liquid treated cultures, indicating that, at the concentrations tested, E-liquid exposure does not substantially alter OMV membrane fluidity, despite observed changes in other OMV characteristics.

A recent review by Zhang et al. (2025) indicates that OMVs from multiple Gram negative oral bacteria carry many different virulence factors and attack nearby host tissues (Zhang et al., 2025). It is possible that although cinnamon and menthol exposure suppresses overall bacterial growth and vesicle output, the OMVs that are produced may be enriched in virulence-associated proteins and DNA, potentially altering the host-pathogen interaction. Zhang and coworkers (2025) also describe that OMVs play an important role in bacteria-bacteria cooperation, extracellular polysaccharide production, and coaggregation, all of which enhances biofilm formation (Zhang et al., 2025). Moreover, since periodontal disease starts with a lack of balance among the oral bacteria communities in biofilms, leading to aberrant host-bacteria interactions, *A. actinomycetemcomitans* behavior after exposure to ECIGs may also change, which may impact the rest of the microbial biofilm community. To address this, *in vitro* studies with continuous flow (open system) multi-species biofilms exposed to E-liquids or ECIG-generated aerosols need to be performed, followed by multi-omics analyses, including meta-transcriptomes, meta-proteomes and metabolomes. Extraction of OMVs from biofilms is possible, but the number of OMVs that can be collected from these systems is experimentally limiting. We therefore chose to investigate the impact of E-liquid exposure on planktonic cells to enable controlled and reproducible comparisons. Other future studies may also focus on other species, members of the red complex, including *Porphyromonas gingivalis*, *Tanarella forthysia* and *Treponema denticola* (Suzuki et al., 2013). Despite these limitations, the comparative experimental design supports the reported trends. Future work will probe the specific alterations in more detail.

Taken together, our findings indicate that flavored E-liquids, particularly cinnamon, can alter both the quantity and composition of *A. actinomycetemcomitans* OMVs. These changes could have important consequences for both bacteria-bacteria and host–bacteria interactions, including the potential for enhanced toxin-mediated pathogenicity despite reduced bacterial proliferation.

## Data Availability

The raw data supporting the conclusions of this article will be made available by the authors, without undue reservation.
